# The evolution of sexes: A specific test of the disruptive selection theory

**DOI:** 10.1002/ece3.3656

**Published:** 2017-11-26

**Authors:** Jack da Silva

**Affiliations:** ^1^ School of Biological Sciences University of Adelaide Adelaide SA Australia

**Keywords:** anisogamy, disruptive selection theory, gamete competition theory, isogamy, oogamy, sexes

## Abstract

The disruptive selection theory of the evolution of anisogamy posits that the evolution of a larger body or greater organismal complexity selects for a larger zygote, which in turn selects for larger gametes. This may provide the opportunity for one mating type to produce more numerous, small gametes, forcing the other mating type to produce fewer, large gametes. Predictions common to this and related theories have been partially upheld. Here, a prediction specific to the disruptive selection theory is derived from a previously published game‐theoretic model that represents the most complete description of the theory. The prediction, that the ratio of macrogamete to microgamete size should be above three for anisogamous species, is supported for the volvocine algae. A fully population genetic implementation of the model, involving mutation, genetic drift, and selection, is used to verify the game‐theoretic approach and accurately simulates the evolution of gamete sizes in anisogamous species. This model was extended to include a locus for gamete motility and shows that oogamy should evolve whenever there is costly motility. The classic twofold cost of sex may be derived from the fitness functions of these models, showing that this cost is ultimately due to genetic conflict.

## 1 INTRODUCTION

Gamete dimorphism, or anisogamy, in the extreme form of small motile sperm and large nonmotile eggs, or oogamy, defines sexes and is characteristic of plants and animals. An elegant and enduring theory for the evolution of anisogamy from gamete monomorphism, or isogamy, is that as body size increases there is selection for a larger zygote to store nutrients used in early development, which in turn selects for larger gametes. This may provide the opportunity for one mating type to produce more numerous, smaller gametes, thereby increasing its fertility via competition with other gametes of the same mating type, and thus forcing the other mating type to produce fewer, larger gametes (Bell, 1978; Bulmer, 1994; Charlesworth, 1978; Maynard Smith, 1978, 1982; Parker, Baker, & Smith, 1972). Therefore, this theory proposes competition among gamete size alleles within mating types and genetic conflict between mating types. As a result, models based on this theory are known as disruptive selection, or gamete competition, models to distinguish them from models based on gamete limitation or intracellular conflicts (see reviews by Randerson & Hurst, 2001b; Lessells, Snook, & Hosken, 2009; Togashi & Cox, 2011).

Two predictions from the disruptive selection theory have been partially upheld: that with an increase in structural complexity or adult body size there is an increase in both zygote size and gamete dimorphism. These predictions have been tested in the algae, which exhibit considerable interspecific variation in structural complexity, body size, and gamete dimorphism (Bell, 1978, 1985; Knowlton, 1974; Madsen & Waller, 1983; Randerson & Hurst, 2001a). However, there are many exceptions to these associations, such as unicellular species that are oogamous and multicellular species that are isogamous, and this has raised doubts about the completeness of the theory (Bell, 1978; Madsen & Waller, 1983). In addition, these predictions may not be specific to the disruptive selection theory. A positive correlation between zygote size and adult size or complexity may be required by the theory to explain the increase in zygote size that leads to the conditions for disruptive selection. However, an increase in zygote size, necessitating fewer, larger gametes, may also result in gamete limitation. If one mating type producing a large nonmotile gamete and the other producing a small motile gamete increase the gamete encounter rate, as proposed in some models of gamete limitation (Cox & Sethian, 1985; Dusenbery, 2000, 2006; Lehtonen & Kokko, 2011), then gamete dimorphism will increase with adult size if zygote size increases with adult size. Therefore, the disruptive selection theory has yet to be rigorously tested. Here, a novel specific prediction is derived from the most thoroughly developed model of the disruptive selection theory available and tested with data from the volvocine algae.

**Figure 1:**
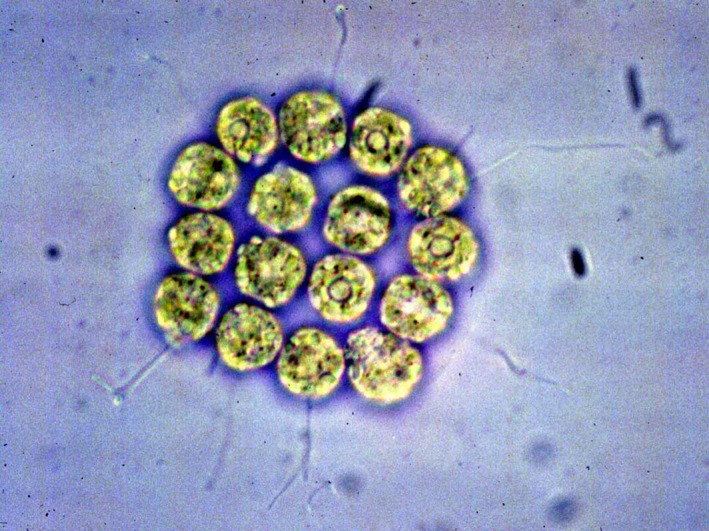
*Gonium pectorale*, a colonial isogamous volvocine alga. Photograph credit: Environmental Protection Agency—http://www.epa.gov/glnpo/image/viz_nat6.html

The volvocine algae (order Chlamydomonadales; formerly Volvocales) (Figure 1) have been particularly useful in testing the disruptive selection theory (Bell, 1978, 1985; Knowlton, 1974; Madsen & Waller, 1983; Randerson & Hurst, 2001a). This group ranges in complexity from unicellular to colonial to multicellular with complete separation between soma and germ, and all three structural grades exhibit both isogamy and some form of anisogamy (Hallmann, 2011). The intensely studied unicellular, isogametic *Chlamydomonas reinhardtii*, is often used to represent the ancestral condition. Colony development in this species is easily selected in the laboratory (Ratcliff et al., 2013) and may have a simple genetic basis (Hanschen et al., 2016; Nedelcu & Michod, 2006). And gamete dimorphism in *C. reinhardtii* may be simulated in the laboratory by allowing gametogenesis to occur via either intracellular transformation, producing a single large gamete, or via one or two mitotic divisions, producing two or four small gametes (Wiese, Wiese, & Edwards, 1979). This mirrors gametogenesis in the multicellular, oogametic *Volvox carteri*, in which eggs develop directly from large reproductive cells and sperm are the product of seven sequential mitotic divisions, resulting in 128 sperm per reproductive cell (Hallmann, Godl, Wenzl, & Sumper, 1998). Interestingly, the genetic loci specifying mating type and gamete size in *V. carteri* have evolved close physical linkage, as inferred by comparison with the homologous loci in *C. reinhardtii*, which are not closely linked (Ferris et al., 2010), and are coregulated (Geng, de Hoff, & Umen, 2014). The evolution of tight linkage between mating type and gamete size loci is predicted by a population genetic formulation of the disruptive selection theory (Charlesworth, 1978).

Here, a novel prediction specific to the disruptive selection theory is derived from an existing game‐theoretic model that represents the most complete model of the theory available. This prediction is tested with data from the volvocine algae, including recently described species. Bulmer and Parker (2002) extended the game‐theoretic model of the disruptive selection theory of Maynard Smith (1978, 1982) to include explicit functions for gamete and zygote survival or viability. Making these functions explicit allows predictions of the conditions leading to evolutionarily unstable isogamy and ensuing evolutionarily stable anisogamy. Furthermore, the parameterization of this model makes it easy to estimate the parameters using few assumptions and thus test these predictions. It is shown here that anisogamous species are predicted to always have an anisogamy ratio greater than 3. This is upheld for the species analyzed. The model and predictions are then confirmed using a fully population genetic implementation. Extending the population genetic model to include a locus for motility predicts the evolution of oogamy in anisogamous species whenever there is costly motility. Finally, using the fitness functions from the model to calculate the fitness “cost of sex” for anisogamous species shows that the cost of sex increases with the degree of anisogamy, reaching the classic twofold cost with oogamy.

## 2 GAME‐THEORETIC MODELS AND PREDICTIONS

In the game‐theoretic model of Bulmer and Parker (2002), the fitness of a new haploid mutant producing gametes of size *m*_1_ in a population of individuals producing gametes of size *m*_2_ is

$$w_{1}\left(m_{1},m_{2}\right)=\frac{M}{m_{1}}g\left(m_{1}\right)f\left(m_{1}+m_{2}\right),$$

where *M* is the fixed budget for gamete production and *g* and *f* are functions for the probability of gamete and zygote survival, respectively. Predictions are derived from this model using two forms of survival function used by Bulmer and Parker.

With what Bulmer and Parker term the “Vance survival function,” which is actually a modification of Vance's function (Vance, 1973) proposed by Levitan (2000)

g(m)=exp(−α/m),

f(S)=exp(−β/S),

where *S* = *m*_1_ + *m*_2_, the zygote size, and α and β are positive scaling parameters. This function is sigmoidal, accelerating from the origin until *m* = α/2 (*S* = β/2) and then decelerating and reaching an asymptotic value of one. For an anisogamous species, the ancestral condition is likely to have been α ≈ β and then become β > α, selecting for a larger zygote (Bulmer & Parker, 2002). Under this model, with isogamy, the optimal isogamete size is $m^{*}=\alpha +\beta /4$, and isogamy is continuously evolutionarily stable when β < 4α. When β > 4α, isogamy becomes evolutionarily unstable and anisogamy becomes continuously evolutionarily stable. With anisogamy, if gamete sizes are *a* and *b* and *a* < *b*,* a* = α + ε and *b* = β – α − ε, where ε is a small deviation that tends to zero as β increases relative to α. Therefore, with large β, α ≈ *a* and β ≈ *a* + *b*. Then, because anisogamy is evolutionary stable when β > 4α, the anisogamy ratio for anisogamous species is predicted to be *b*/*a* > 3.

In a second model, Levitan's survival function is used for zygotes, but gametes do not survive below threshold size δ and have a probability of survival of one above that size. This is the implicit gamete survival function used by Maynard Smith (1978, 1982). With this model, $m^{*}=\beta /4$ and isogamy are continuously evolutionarily stable when β<4δ. When β>4δ, isogamy becomes evolutionarily unstable, and anisogamy becomes continuously evolutionarily stable. Then, with anisogamy, δ = *a* because microgametes always evolve to the smallest viable size, and β ≈ δ + *b*, for large β. Therefore, as in the case with Levitan's survival functions, with large β, the anisogamy ratio for anisogamous species is predicted to be *b*/*a* > 3.

Symbols used for variables, functions, and parameters in these models are defined in Table 1.

**Table 1:** Symbol definitions for the Bulmer and Parker game‐theoretic models

Symbol	Definition
Variables	
*w*	Fitness of a haplotype
*m*	Gamete size
*a*	Microgamete size
*b*	Macrogamete size
*S*	Zygote size
Functions	
*g*(*m*)	Gamete survival function
*f*(*S*)	Zygote survival function
Parameters	
*M*	Fixed budget for gamete production
α	Positive scaling parameter of *g*(*m*)
β	Positive scaling parameter of *f*(*S*)
δ	Threshold size for gamete survival

## 3 METHODS

### 3.1 Data

Gamete volumes, *a* and *b*, were obtained from the literature for each species of the volvocine algae for which the relevant data are available. The volume of a reproductive cell was used as an estimate of the gamete budget, *M*. A reproductive cell refers to a cell that produces gametes, either through direct transformation or through mitotic cell divisions. This may be a zoospore (vegetative cell) of a unicellular or colonial species or a specialized (germ) cell in multicellular species.

Cell volumes were either reported in the literature or calculated from the cell radius if cells were approximately spherical. Most cells are prolate spheroids or spindle‐shaped ellipsoids, whose volume is calculated as (4/3)π*r*_1_ *r*_2_ *r*_3_, where *r*_1_, *r*_2_, and *r*_3_ are the three radii. Typically, the length and width of a cell are reported; the third dimension was assumed to be equal to the width. Often, a range of values is given for each dimension; in these cases, the largest values were used to ensure that mature cells were used, following Randerson and Hurst (2001a). In some cases, cell dimensions were measured from high‐quality drawings or micrographs. Because of inherent errors in estimating volume, especially of gametes (because they are often smaller than reproductive cells) and because gametes are the result of 0 or more cell divisions, macrogamete, or isogamete, volume was adjusted so that the ratio *M*/*b* is the nearest power of 2. In some anisogamous and oogamous species, the macrogamete or egg develops directly from a reproductive cell, and microgametes or spermatozoids are produced by several sequential mitotic divisions of a reproductive cell. For these species, microgamete volume was calculated as the volume of a reproductive cell divided by the number of microgametes. The protoplasmic volume was used as a measure of adult size; this is the sum of the volumes of the individual's cells making up an adult (Randerson & Hurst, 2001a). Data are from Randerson and Hurst (2001a) and references therein, and from references in Harris (2009). In addition, each genus in each family of the Chlamydomonadales, as listed in the online database AlgaeBase (Guiry & Guiry, 2017), was used as an online bibliographic search term, providing additional species for which the required data were available (Demchenko & Mikhailiuk, 2012; Hayama, Nakada, Hamaji, & Nozaki, 2010; Hoham et al., 2006; Ivan & Katya, 2014; Kaštovský, 2008; Kugrens, Clay, & Aguiar, 2000; Ling, 2001; Nováková, 2005; Nozaki & Itoh, 1994; Nozaki, Yamada, Takahashi, Matsuzaki, & Nakada, 2014; Suda, Nozaki, & Watanabe, 2005). The required data were available for a total of 45 species of the Chlamydomonadales (Table 2).

**Table 2:** Structural grade, gamete dimorphism, and size data for volvocine algae

Species	No. of cells	Structural grade	Gamete dimorphism^a^	Protoplasmic volume (μm^3^)	Reproductive cell volume (μm^3^), *M*	Macro‐ or isogamete volume (μm^3^), *b*	Micro‐ or isogamete volume (μm^3^), *a*	Inferred zygote volume (μm^3^), *S*	*b*/*a*
*Carteria palmata*	1	Unicellular	Isogamy	5,080	5,080	5,080	5,080	10,160	1
*Chlamydomonas allensworthii*	1	Unicellular	Anisogamy	950	950	950	59	1,010	16
*Chlamydomonas chlamydogama*	1	Unicellular	Isogamy	1,120	1,120	560	560	1,120	1
*Chlamydomonas indica*	1	Unicellular	Isogamy	733	733	183	183	367	1
*Chlamydomonas microhalophila*	1	Unicellular	Isogamy	1,508	1,508	754	754	1,508	1
*Chlamydomonas moewusii*	1	Unicellular	isogamy	8,370	8,370	2,093	2,093	4,185	1
*Chlamydomonas reinhardtii*	1	Unicellular	Isogamy	11,000	11,000	5,500	5,500	11,000	1
*Chlamydomonas sphagnicola*	1	Unicellular	Oogamy	3,591	3,591	3,591	225	3,816	16
*Chlamydomonas suboogama*	1	Unicellular	Anisogamy	3,054	3,054	3,054	191	3,245	16
*Chlorogonium mariae*	1	Unicellular	Isogamy	524	524	131	131	262	1
*Chloromonas chenangoensis*	1	Unicellular	Isogamy	4,009	4,009	4,009	4,009	8,018	1
*Lobocharacium coloradoense*	1	Unicellular	Isogamy	1,734,159	1,734,159	524	524	1,047	1
*Lobochlamys segnis*	1	Unicellular	Isogamy	700	700	22	22	44	1
*Oogamochlamys ettlii*	1	Unicellular	Oogamy	6,842	6,842	6,842	428	7,270	16
*Oogamochlamys gigantea*	1	Unicellular	Oogamy	8,181	8,181	8,181	128	8,309	64
*Oogamochlamys zimbabwiensis*	1	Unicellular	Oogamy	4,189	4,189	4,189	262	4,451	16
*Pseudocarteria corcontica*	1	Unicellular	Isogamy	13,360	13,360	13,360	13,360	26,719	1
*Tetrabaena socialis*	4	Colonial	Isogamy	11,800	2,950	738	738	1,475	1
*Stephanosphaera pluvialis*	8	Colonial	Isogamy	10,648	1,331	83	83	166	1
*Desmotetra antarctica*	16	Colonial	Isogamy	147,244	9,203	575	575	1,150	1
*Desmotetra aureospora*	16	Colonial	Isogamy	77,584	4,849	303	303	606	1
*Gonium maiaprilis*	16	colonial	Isogamy	28,272	1,767	221	221	442	1
*Gonium pectorale*	16	Colonial	Isogamy	75,600	4,725	2,363	2,363	4,725	1
*Gonium quadratum*	16	Colonial	Isogamy	11,200	700	697	697	1,394	1
*Gonium viridistellatum*	16	Colonial	Isogamy	28,300	1,769	884	884	1,769	1
*Volvulina pringsheimii*	16	Colonial	Isogamy	28,300	1,769	1,767	1,767	3,534	1
*Colemanosphaera charkowiensis*	32	Colonial	Anisogamy	82,318	2,572	2,572	80	2,653	32
*Eudorina elegans*	32	Colonial	Anisogamy/internal	232,000	7,250	3,625	57	3,682	64
*Gonium multicoccum*	32	Colonial	Isogamy	97,700	3,053	3,053	3,053	6,106	1
*Pandorina morum*	32	Colonial	Isogamy	294,000	9,188	574	574	1,148	1
*Platydorina caudata*	32	Colonial	Anisogamy	262,000	8,188	4,094	128	4,222	32
*Volvulina steinii*	32	Colonial	Isogamy	70,600	2,206	2,206	2,206	4,413	1
*Pleodorina indica*	64	Multicellular	Anisogamy/internal	357,000	9,200	1,150	18	1,168	64
*Astrephomene gubernaculifera*	128	Multicellular	Isogamy	6,100,000	48,800	3,050	3,050	6,100	1
*Pleodorina japonica*	128	Multicellular	Anisogamy/internal	1,040,000	14,100	14,100	110	14,210	128
*Volvox pocockiae*	1,500	Multicellular	Oogamy	2,650,000	113,000	113,000	1,766	114,766	64
*Volvox tertius*	2,000	Multicellular	Oogamy	763,000	655,000	655,000	20,469	675,469	32
*Volvox carteri*	3,000	Multicellular	Oogamy	1,570,000	10,300	10,300	80	10,380	128
*Volvox aureus*	3,200	Multicellular	Oogamy	362,000	7,230	7,230	226	7,456	32
*Volvox obversus*	4,000	Multicellular	Oogamy	2,090,000	38,800	38,800	303	39,103	128
*Volvox africanus*	6,000	Multicellular	Oogamy	963,000	47,700	47,700	373	48,073	128
*Volvox capensis*	20,000	Multicellular	Oogamy	42,100,000	125,000	125,000	244	125,244	512
*Volvox globator*	22,000	Multicellular	Oogamy	737,000	11,000	11,000	43	11,043	256
*Volvox dissipatrix*	31,800	Multicellular	Oogamy	133,000,000	33,500	33,500	131	33,631	256
*Volvox rousseletii*	50,000	Multicellular	Oogamy	105,000,000	14,100	14,100	28	14,128	512

a Anisogamy/internal refers to anisogamy with internal fertilization.

### 3.2 Phylogenetic analysis

**Figure 2:**
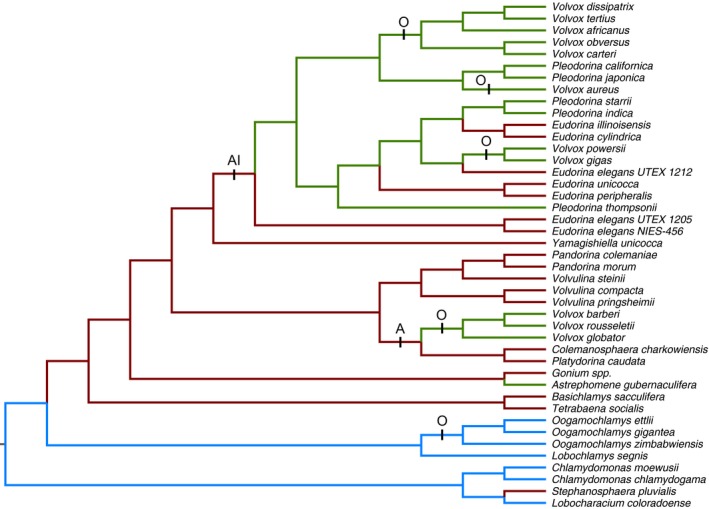
Origins of structural grades and gamete dimorphisms mapped onto a composite phylogeny of the volvocine algae. Blue branches represent unicellular lineages, red branches represent colonial lineages, and green branches represent multicellular lineages. Bars indicate gamete dimorphism: A, anisogamy; AI, anisogamy with internal fertilization; O, oogamy. Isogamy is assumed to be ancestral

Origins of structural grades and gamete dimorphisms were mapped on to a composite phylogeny constructed to include as many of the species in Table 2 as possible (27 species) (Figure 2). Multicellularity is defined as the structural grade in which some cells are somatic. The composite phylogeny was constructed from an exhaustive 18S rRNA Bayesian phylogenetic analysis of the Chlamydomonadales (Volvocales) (Nakada, Misawa, & Nozaki, 2008), with the “Reinhardtinia” of this large phylogeny replaced with a higher resolution phylogeny of the three families Tetrabaenaceae, Goniaceae, and Volvocaceae. This latter phylogeny, which includes most of the colonial and multicellular species in Table 2, is based on a Bayesian analysis of five chloroplast genes (Nozaki et al., 2014). A third phylogeny of the volvocine algae, also based on a Bayesian analysis of the same five chloroplast genes (Herron & Michod, 2008), and which is consistent with the phylogeny of Nozaki et al. (2014), was used to place *Astrephomene gubernaculifera* as a sister taxon to the *Gonium* clade. The composite phylogeny contains 27 species from Table 2, and all remaining species from the Nozaki et al. (2014) phylogeny for which structural grade and gamete dimorphism have been reported (Goldstein, 1964; Nozaki & Kuroiwa, 1990, 1991, 1992; Nozaki, Ott, & Coleman, 2006; Pocock, 1955; Smith, 1944; Stein, 1959; Vande Berg & Starr, 1971). The additional 15 taxa from the Nozaki et al. (2014) phylogeny were included to provide additional resolution to the intensely studied Volvocaceae, which are all colonial or multicellular and vary substantially in gamete dimorphism.

The most parsimonious origins of structural grades and gamete dimorphisms were mapped on to the phylogeny, assuming that unicellular isogamy is ancestral (Figure 2). These mappings are mostly unambiguous. One of two exceptions is the origin of multicellularity from colonial ancestors in the clade defined by *Eudorina* spp. and allied taxa in the genera *Volvox* and *Pleodorina*, for which either four independent origins or one origin followed by three losses is equally parsimonious. A single origin is used to maintain consistency with the more detailed study of the evolution of multicellularity by Herron, Hackett, Aylward, and Michod (2009). The three origins of multicellularity in the entire tree are supported by each multicellular clade exhibiting a distinct form of development (Hallmann, 2006; Yamashita et al., 2016). The other exception to unambiguous mappings is the origin of oogamy in the clade containing *Volvox tertius* and *Volvox aureus*. Two independent origins of oogamy within the clade, or one origin in the ancestor and a subsequent loss, are equally parsimonious. Two independent origins of oogamy in this clade are assumed on the basis that reversions from oogamy appear to be rare, given that all extant plants and animals are oogamous.

### 3.3 Population genetic model

A population genetic implementation of the Bulmer and Parker (2002) game‐theoretic model described above was developed to numerically simulate the evolution of gamete size in the volvocine algae with realistic mutation patterns, genetic drift, and selection. The model was used to verify the game‐theoretic model and the use of the large‐β approximation for estimating α and β. A gamete size locus is physically linked to a mating type locus and determines the number of gametogenic mitoses. Allele *i* at this locus produces gametes of size *m*_*i*_ = *M*/2^*i*^, where *i* is the number of gametogenic mitoses, ranging from 0 to an assigned maximum value, *d*. The fitness of an allele depends on the gamete's mating type because the mean viability of zygotes to which the gamete contributes depends on gamete size allele frequencies in the opposite mating type. For example, using the Levitan equation for gamete survival, the fitness of allele *i* in mating type plus (+) is

$$w_{i}^{+}=\frac{M}{m_{i}}g\left(m_{i}\right)\sum_{j=0}^{d}f\left(m_{i}+m_{j}\right)x_{j}^{-},$$

where $x_{j}^{-}$ is the frequency of gamete size allele *j* in mating type minus (−). Mutation is symmetrical and stepwise within a mating type. For example, gamete size allele 0 mutates with probability μ/2 per generation to allele 1, and allele 1 mutates with probability μ/2 to allele 0 and with probability μ/2 to allele 2.

Changes in allele frequencies after selection, mutation, and genetic drift were tracked using recursion equations. Selection occurs within mating types, as gametes within a mating type compete for fusion with gametes of the opposite mating type. Therefore, the frequency of a gamete size allele on a mating type plus chromosome after selection is

$$y_{i}^{+}=x_{i}^{+}\frac{w_{i}^{+}}{{\overset{¯}{w}}^{+}},$$

where $x_{i}^{+}$ is the current frequency and ${\overset{¯}{w}}^{+}$ is the mean fitness of mating type plus. Genetic drift was simulated by the random sampling of allele counts from a multinomial distribution, with probabilities of success equal to allele frequencies and the number of trials equal to the population size *N*. Genetic drift was necessary for the evolution of the disassortative fusion of dimorphic gametes because one mating type must become statistically linked to one gamete size (Charlesworth, 1978). Genetic drift was applied to each mating type separately to maintain a “sex ratio” of one. Simulations were initialized with macrogamete isogamy, that is, with both gametes being produced by the direct transformation of a reproductive cell, and with μ = 10^−3^ and *N* = 10^4^, and were run for 10^4^ generations. Other values for these parameters gave the same results. The remaining parameters, *M*, α, and β, were estimated from each anisogamous species of volvocine algae, with α = *a* and β = *S*. The maximum number of gametogenic mitoses was set to 9, the maximum observed for the volvocine algae in this dataset. The primary output from a simulation was the gamete size for each mating type.

### 3.4 Modeling oogamy

The population genetic model was extended to include a locus for gamete motility to allow the evolution of oogamy, in which the microgamete is motile and the macrogamete is nonmotile, as distinct from anisogamy, in which both gametes are motile. Motility was assumed to require a proportion of a gamete's volume, *p*, so that its facultative size, which affects its viability and contributes to the viability of a zygote, is *m*(1 − *p*). This cost of motility is assumed to be proportional to gamete size because the amount of energy and materials required to move a gamete are expected to increase with its size. Nonmotile gametes were assumed to fuse only with motile gametes because of negligible encounter rates between nonmotile gametes in the volvocine algae. Zygote size was calculated from the sum of the facultative sizes of fusing gametes. The fitness of a haplotype (mating type, gamete size, and motility) therefore depended not only on gamete size, but also on whether it was motile; the zygote viability component of fitness for a *nonmotile* gamete depends on the sizes of *motile* gametes of the opposite mating type. As before, mutation was symmetrical and stepwise within mating types: Mutation occurred among gamete size alleles within motility types (motile, nonmotile), and between motility types for each gamete size. Simulations were initialized with the largest, motile gametes possible for the species. Initializing simulations with isogametes of other sizes gave the same results. The parameters α and β were estimated from gamete sizes, as before, but were adjusted to account for the reduced facultative size of the microgamete due to motility.

## 4 RESULTS

### 4.1 Zygote volume

**Figure 3:**
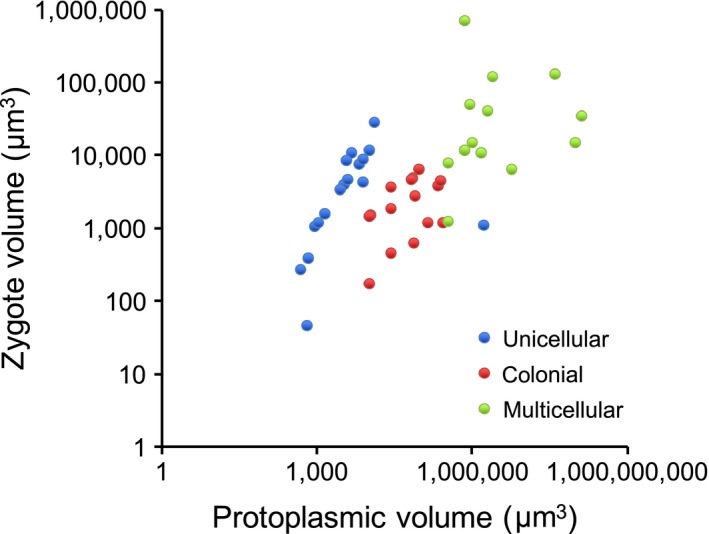
Estimated zygote volume as a function of protoplasmic volume for volvocine algae

A general prediction of the disruptive selection theory previously confirmed for the volvocine algae, that zygote (or macrogamete) size increases with adult size (Bell, 1985; Randerson & Hurst, 2001a), is upheld for the 45 species analyzed here (Figure 3). Only 27 of the 45 species are represented in the phylogeny (Figure 2), and therefore, no attempt was made to control for phylogeny. However, it is clear that zygote volume increases with protoplasmic volume, as previously shown (Randerson & Hurst, 2001a). Zygote volume also increases with structural grade, going from colonial to multicellular forms, but this obviously because multicellular forms are larger. The relationship for unicellular forms appears distinct from that of the other structural grades. This may be simply because the protoplasmic volume is also the reproductive cell volume for unicellular species, whereas the reproductive cell volume for colonial and multicellular species is necessarily smaller than the protoplasmic volume. An apparent departure from the zygote‐protoplasmic volume relationship for unicellular species is due to the very large multinucleate unicellular *Lobocharacium coloradoense*, which is effectively colonial.

### 4.2 Gamete dimorphism

Another general prediction of the disruptive selection theory previously upheld is that gamete dimorphism increases with structural complexity or adult size (Bell, 1978, 1985; Knowlton, 1974; Madsen & Waller, 1983; Randerson & Hurst, 2001a). Species in all three structural grades exhibit both isogamy and some form of anisogamy, although multicellular species are predominantly oogamous (Table 2). Exceptions to the pattern of unicellular species being isogamous are *Chlamydomonas allensworthii* and *Chlamydomonas suboogama*, which are anisogamous, and *Chlamydomonas sphagnicola* and members of *Oogamochlamys*, which are oogamous. Colonial species, with colonies containing 4–32 cells, exhibit isogamy and anisogamy. Multicellular species, containing 64–50,000 cells, are anisogamous or oogamous, except for *A. gubernaculifera*, which is isogamous.

Mapping the origins of coloniality and multicellularity and the origins of anisogamy and oogamy on to a phylogeny of the volvocine algae emphasizes the lack of a simple relationship between structural complexity and gamete dimorphism. Using a composite phylogeny for the species analyzed (Table 2) and additional species in the Tetrabaenaceae, Goniaceae, and Volvocaceae (TGV clade) indicates that coloniality evolved at least twice among these taxa, once in the *Stephanosphaera pluvialis* lineage and once in the ancestor of the TGV clade (Figure 2). These latter taxa include most of the colonial and multicellular species analyzed (Table 2). Multicellularity evolved at least three times in the TGV clade from a colonial ancestor: once in the *Astrephomene* lineage, once in the clade containing *Volvox globator,* and once in the clade containing the remaining *Volvox* species plus *Pleodorina* and *Eudorina*. These origins of multicellularity are consistent with a previous study (Herron et al., 2009) and are supported by having different developmental patterns (Hallmann, 2006; Yamashita et al., 2016). Anisogamy evolved at least twice in this phylogeny, once in the lineage leading to clade including the colonial *Platydorina caudata* and the related multicellular *Volvox* species, and once in the lineage leading to the colonial *Eudorina* and the related multicellular *Pleodorina* and *Volvox*. In the latter case, anisogamy is coupled with internal fertilization. Interestingly, in both cases, the phylogeny suggests that anisogamy evolved before multicellularity. Oogamy is estimated to have evolved at least five times in this phylogeny, once in the unicellular *Oogamochlamys*, apparently from an isogamous ancestor, once from anisogamy in the multicellular *V. globator* clade, and three times from anisogamous ancestors with internal fertilization in the clades containing the remaining *Volvox* species. This phylogeny suggests only a weak association between gamete dimorphism and structural complexity. While both origins of anisogamy occurred in colonial lineages, other colonial lineages remained isogamous. Similarly, while oogamy originated independently in the four multicellular *Volvox* clades, multicellular *Pleodorina* remained anisogamous, as did multicellular *Astrephomene*. And finally, the unicellular *Oogamochlamys* evolved oogamy.

### 4.3 Anisogamy ratio

**Figure 4:**
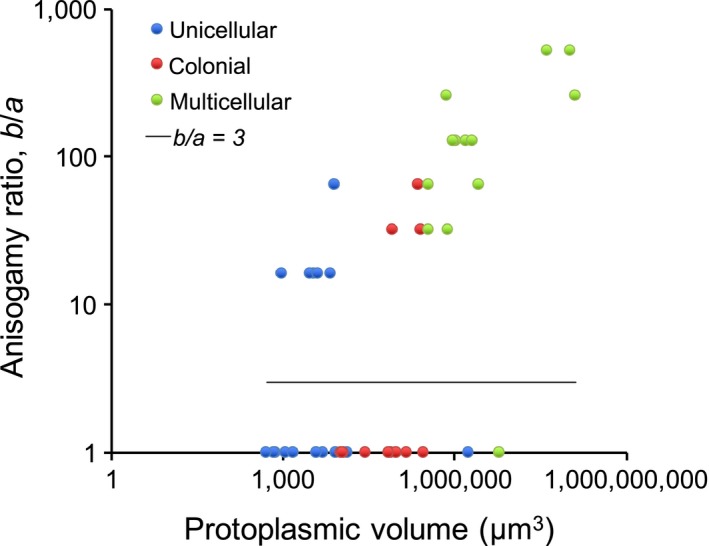
Anisogamy ratio plotted against the protoplasmic volume for volvocine algae. The horizontal line indicates the predicted “anisogamy threshold”

Isogamy exists across all three structural grades and across a wide range of protoplasmic volumes (Figure 4). However, a positive correlation between the anisogamy ratio and protoplasmic volume and structural grade is apparent for anisogamous species. Therefore, what is missing is an understanding of why some species are anisogamous, while others with the same structural complexity and size are isogamous. An explanation may come partly from the model of Bulmer and Parker (2002), which shows that for sigmoidal gamete and zygote survival functions, anisogamy is continuously evolutionarily stable when β>4α. Thus, ecological conditions that increase β or decrease α may lead to anisogamy independently of adult size or complexity (Bulmer & Parker, 2002) (see Section 5). This prediction is shown here to be equivalent to an anisogamy ratio *b*/*a* > 3, which is confirmed for all 21 anisogamous (including oogamous) species; for these species, the anisogamy ratio clusters well above 3 (Figure 4).

### 4.4 Population genetic verification and oogamy

For the population genetic model, parameters (*M*, β, α) were estimated for each anisogamous species (β and α could be estimated only for anisogamous species) and used to simulate gamete size evolution, starting from isogamy. The model accurately predicts the evolution of gamete sizes, and thus anisogamy ratios. It should be noted, however, that this is not an independent test of the Bulmer and Parker (2002) model because β and α are estimated from the data, namely the gamete volumes, *a* and *b*. Nevertheless, the population genetic implementation supports the game‐theoretic model and shows that the large‐β approximations of β and α are good. The population genetic implementation also shows that anisogamy could evolve from isogamy by mutation generating variation in gamete size and then genetic drift and selection linking a gamete size to a mating type, resulting in the disassortative fusion of dimorphic gametes.

The population genetic model extended to include gamete motility and with parameters estimated for each anisogamous species in Table 2 predicts the evolution of anisogamy, with both gametes motile, from isogamy for a low cost of motility (a low proportion of gamete size allocated to motility; *p* = .01). With a moderate cost of motility (*p* = .1), pseudooogamy, in which the microgamte is motile and ~80% of macrogamtes are nonmotile, evolves. With a high cost of motility (*p* = .5), oogamy evolves. Therefore, if there is a high proportional cost of motility, the macrogamete, which will bear a greater cost in absolute terms because it is larger, will evolve to be nonmotile, while the microgamete will retain motility to enable gamete contact.

### 4.5 Cost of sex

Estimation of model parameters for anisogamous species allows calculation of the cost of sex due to anisogamy (Maynard Smith, 1978). Using the Levitan survival function for gamete survival, the fitness for an individual of an anisogamous species at equilibrium is the fitness of the macrogamete producer because the number of zygotes produced is limited by the number of macrogametes:

$$w_{b}\left(a,b\right)=\frac{M}{b}g\left(b\right)f\left(S\right).$$

With hypothetical isogamy for the same species, with isogamete size *m* = (*a* + *b*)/2, gamete fitness is

$$w_{m}\left(m,m\right)=\frac{M}{m}g\left(m\right)f\left(S\right).$$

The fold cost of sex is then

$$C=\frac{w_{m}\left(m,m\right)}{w_{b}\left(a,b\right)}=\frac{b}{m}\cdot \frac{g(m)}{g(b)},$$

or, with a gamete survival threshold, *C* = *b*/*m*. With a gamete survival threshold, the cost of sex may also be expressed as a function of the anisogamy ratio:

$$C=\frac{2b/a}{1+b/a}.$$

**Figure 5:**
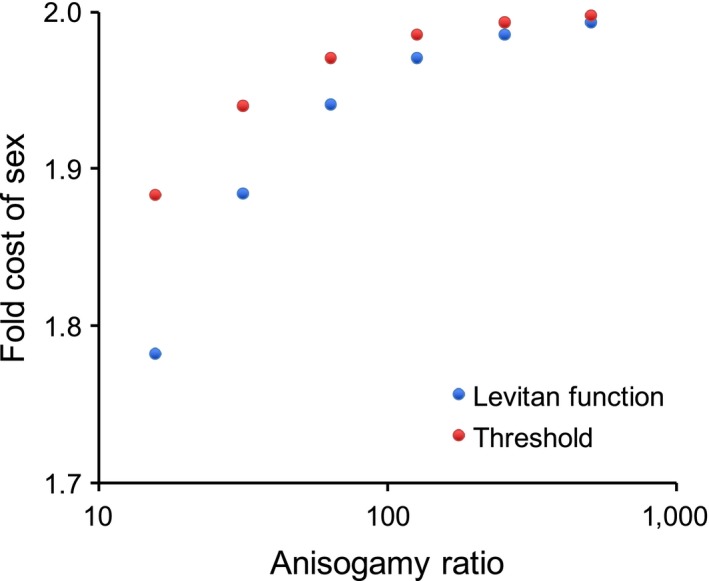
Fold fitness “cost of sex” plotted against the anisogamy ratio for the volvocine algae analyzed. Costs are shown for the Levitan gamete survival function and a gamete survival threshold

The cost of sex approaches two as the anisogamy ratio increases (Figure 5). The cost is more moderate for more moderate forms of anisogamy because the cost of sex is due mainly to the higher fertility with hypothetical isogamy (*M*/*m* > *M*/*b*), and this difference is less extreme with moderate anisogamy (*m* ≈ *b*). The cost is also ameliorated if gamete survival is a continuous function of gamete size, as represented by the Levitan survival function (Figure 5). This is because with the Levitan function macrogametes experience higher survival than isogametes. Thus, the classic twofold cost of sex may be derived from the model's fitness functions. In addition, it is shown here that the cost increases with the degree of anisogamy and that it is greater if gamete viability is a continuous function of gamete size, rather than dependent on a threshold size.

## 5 DISCUSSION

The Bulmer and Parker (2002) game‐theoretic model provides the most complete description of the disruptive selection theory of the evolution of anisogamy available. With sigmoidal survival functions for gametes and zygotes, this model predicts that isogamy becomes evolutionarily unstable, and anisogamy continuously evolutionarily stable, when the survival function scaling parameters have the relation β > 4α. It is shown here that with large β this is equivalent to an anisogamy ratio of *b*/*a* > 3, regardless of whether both gamete and zygote survival are described by the Levitan survival function, or whether there is a size threshold for gamete survival. This prediction is borne out for all 21 anisogamous species of volvocine algae analyzed.

The game‐theoretic approach and the use of the large‐β approximation for estimating β and α were verified using a fully population genetic version of the model, which accurately simulates the evolution of gamete sizes for anisogamous species. This model differs from the population genetic models of Charlesworth (1978) mainly in the forms of the zygote and gamete survival functions. The model developed here uses the sigmoidal (Levitan) or threshold survival functions of Bulmer and Parker (2002), whereas Charlesworth assumed that zygote survival is a monotonically increasing function of zygote size and that gamete survival is independent of size. Consideration of the shape of the survival function suggests that a monotonically increasing function is unrealistic and that a sigmoidal function is plausible (Bulmer, Luttikhuizen, & Parker, 2002; Bulmer & Parker, 2002; Randerson & Hurst, 2001b, 2002). Using the parameterization of Bulmer and Parker (2002) also facilitated parameter estimation, as for large β, α and β can be estimated from gamete sizes.

The explanation for the evolution of anisogamy via disruptive selection has been that larger or more complex organisms require more resources for timely development and growth, which selects for a larger zygote, which in turn selects for larger gametes. As gamete size increases, however, one mating type may increase its fitness by producing more numerous, smaller gametes, even if they have a lower probability of survival than larger gametes. Two predictions from this theory, that both zygote size and gamete dimorphism increase with adult size, have previously been partially supported for the volvocine algae (Bell, 1985; Randerson & Hurst, 2001a). The prediction that zygote size increases with adult size is also supported by the present study. However, with additional data on unicellular species incorporated into the present study, a simple relationship between the anisogamy ratio and adult size is no longer clear. Instead, the anisogamy ratio for anisogamous species clusters well above 3, as predicted from the Bulmer and Parker (2002) model, and for these species the anisogamy ratio increases with adult size.

The disruptive selection theory has in the past been interpreted to predict that unicellular species are isogamous and that multicellular species, because of their greater complexity and larger size, are anisogamous. The exceptions to this pattern have cast doubt on the theory's completeness (Bell, 1978; Madsen & Waller, 1983). Therefore, it is useful to examine the exceptions in this dataset. Of the 17 unicellular species studied here, six exhibit gamete dimorphism. These six species are not larger than the isogamous species (Table 2). However, all six species produce the macrogamete directly from the parent (reproductive) cell, without cell division, by intracellular transformation, while producing microgametes by several gametogenic cell divisions. For the isogamous species, both gametes are often produced by one or more gametogenic mitoses. Clearly, intracellular transformation produces the largest possible macrogamete and thus produces a large zygote. For unicellular species, the benefit of a large zygote may be increased survival of the dormant zygospore (Madsen & Waller, 1983) or rapid postzygotic growth (Bell, 1978). Whether these arguments apply to the species under consideration are not known.

Bulmer and Parker (2002) suggest that unicellular species may evolve anisogamy via a reduction in α, rather than an increase in β. That is, under conditions of rapid fertilization, one of the gametes may be small and need to survive only until fertilization. However, this explanation does not appear to fit the unicellular species analyzed here as anisogamy appears to have evolved by an increase in the size of the macrogamete, rather than a reduction in the size of the microgamete.

All of the multicellular species exhibit anisogamy with internal fertilization or oogamy, except *A. gubernaculifera*, which is isogamous. All the oogamous species produce macrogametes by the intracellular transformation of large reproductive cells, thereby producing large zygotes (Table 2). In contrast, *A. gubernaculifera* gametes are the product of four sequential gametogenic cell divisions. As a result, *A. gubernaculifera* produces one of the smallest zygotes of all the multicellular species analyzed, even though its reproductive cells are larger than those of many of the other multicellular species. Therefore, *A. gubernaculifera* either does not require a large zygote for timely development or requires both gametes to be small and motile, as has been proposed for some marine green algae (Togashi, Bartelt, Yoshimura, Tainaka, & Cox, 2012) (see below). It should be noted that *A. gubernaculifera* did not evolve multicellularity recently, as sterile soma evolved between 134 and 227 million years ago in the lineage leading to *Astrephomene*, thus preceding the evolution of multicellularity in the other lineages studied here (Herron et al., 2009). This implies that there would have been ample time for oogamy to evolve in this lineage if it was closely tied to multicellularity.

The phylogenetic analysis confirms that there is only a weak association between oogamy and multicellularity. Oogamy has evolved in the primitively unicellular *Oogamochlamys* lineage (as well as in the unicellular *C. sphagnicola*, although the phylogenetic position of this species has not been determined), multicellularity has evolved in the primitively isogamous *A. gubernaculifera*, and the multicellular *Pleodorina* have not evolved oogamy. These analyses also show that oogamy is not always preceded by anisogamy, as in the case of the *Oogamochlamys* lineage, although the steps in character evolution may change as species are added to this poorly studied part of the phylogeny. In contrast, oogamy may have evolved independently three times in *Volvox* lineages closely related to *Pleodorina*, having been preceded by the evolution of anisogamy with internal fertilization. This form of anisogamy has been proposed as a route to oogamy in *Volvox* (Nozaki et al., 2014). However, the independent evolution of oogamy in *Volvox* closely allied to *Platydorina* from anisogamy with external fertilization, suggests that this route is not necessary.

The disruptive selection theory has been criticized for ignoring the effects of gamete size on gamete encounter rates under low gamete density or gamete limitation (Cox & Sethian, 1985; Dusenbery, 2000, 2006; Iyer & Roughgarden, 2008). Although gamete limitation may select for anisogamy, its importance is not clear (Lessells et al., 2009; Parker & Lehtonen, 2014). Togashi et al. (2012) describe an individual‐based population genetic model of disruptive selection that also considers gamete collision kinetics. They use this model to explain the evolution of gamete dimorphism in marine green algae living in various environments. For example, they argue that a deep water niche should select for extreme anisogamy, from an ancestral condition of moderate anisogamy, because diminished photosynthesis in deep water selects for a zygote large enough to store sufficient resources for rapid early development. The results presented here suggest that the simpler model of Bulmer and Parker (2002) may be sufficient to explain patterns of gamete dimorphism in the volvocine algae, which have simpler life cycles and inhabit shallow freshwater or moist soil. The parameters of this model could, however, be set to reflect the different environmental conditions specified by Togashi et al. (2012). For example, conditions favoring a large zygote may be reflected in large β. Or, conditions favoring large gametes may be represented by large δ and are expected to result in the “large isogamy” of Togashi et al. (2012) if the optimal gamete size is less than the threshold for gamete viability (m* < δ) (Bulmer & Parker, 2002).

Among the marine green algae listed by Togashi et al. (2012) (their Table S1), 17 of the 18 species in the order Bryopsidales, which generally produce large zygotes (with presumably large β), have anisogamy ratios that are >2.5, and generally much higher, consistent with the prediction derived from the Bulmer and Parker (2002) model. However, some members of other orders exhibit slight anisogamy with anisogamy ratios ≤2. Togashi et al. (2012) explain slight anisogamy as the result of microgametes evolving a phototactic organ (eyespot), which limits their minimum size. It is argued that in these species eyespots evolved in both gametes because these allowed them to move to the two‐dimensional water surface, where they increase their encounter rates compared to the three‐dimensional water column. However, if it can be assumed that the volume occupied by the phototactic organ does not contribute to gamete or zygote survival, then subtracting this volume from both gametes would increase the anisogamy ratio with respect to the facultative volumes of the gametes, because the organ represents a larger proportion of the volume of the smaller gamete. Thus, the evolution of a phototactic organ in microgametes appears to explain slight anisogamy in a manner consistent with the Bulmer and Parker (2002) model. Interestingly, the isogametes of the multicellular *A. gubernaculifera* possess eyespots (Stein, 1958).

Bulmer and Parker (2002) also consider the case of a minimum volume for a gamete, required, for example, for chromosomes or a phototactic organ, but which does not contribute to the facultative volume affecting viability. In this case, they show that isogamy may be continuously evolutionarily stable for β ≫ 4α, perhaps explaining isogamy in large and complex species of volvocine algae (Figure 4).

Extending the population genetic version of the Bulmer and Parker (2002) model developed here to include a locus for gamete motility shows that oogamy is expected to evolve from isogamy when there is a high cost of motility. The explanation is simply that the proportional gain in facultative size to large gametes from a loss of motility is greater than the gain by small gametes, but one gamete type must remain motile to ensure fusion. This is similar to the explanation offered by Parker et al. (1972). Other hypotheses for the evolution of oogamy, reviewed by Parker (2011) and Lessells et al. (2009), have focused on a presumed increase in swimming speed of smaller gametes and an increase in target area of larger gametes. The inclusion of motility in the population genetic model developed here shows that a simple consideration of the effects of gamete sizes on viabilities may be sufficient to explain the evolution of oogamy. However, nonoogamous anisogamy may be maintained even with costly motility if motility is necessary in both gametes, for example, to allow both gametes to reach the water surface and thereby increase encounter rates (Togashi et al., 2012).

When the disruptive selection theory was first proposed, anisogamy was interpreted as parasitism of macrogamete producers (females) by microgamete producers (males), or as the original conflict between the sexes (Parker et al., 1972). The theory lends itself well to such game theory analysis, in which the evolution of anisogamy is the outcome of the susceptibility of isogamy to invasion by microgametes (Bulmer, 1994; Bulmer & Parker, 2002; Matsuda & Abrams, 1999; Maynard Smith, 1978, 1982). In this context, the cost of sex due to anisogamy (Maynard Smith, 1978), shown here to be consistent with the theory, may be viewed as ultimately the result of genetic conflict. In addition, the cost of sex is shown to depend on the degree of anisogamy and the relationship between gamete viability and gamete size.

Previous studies have generally upheld the common predictions of gamete competition and gamete limitation models, that zygote size and gamete dimorphism increase with adult size or complexity, although noting many exceptions. Here, the prediction that zygote size increases with adult size is confirmed for the volvocine algae, but gamete dimorphism is found to have more complex relationships with adult size and structural complexity. The pattern of gamete dimorphism is partially explained by a prediction specific to the disruptive selection theory that the anisogamy ratio is greater than three for anisogamous species. Consideration of restrictions to the minimal gamete size due to obligate components that do not affect viability may help explain isogamy in large and structurally complex species.

## CONFLICT OF INTEREST

None declared.
